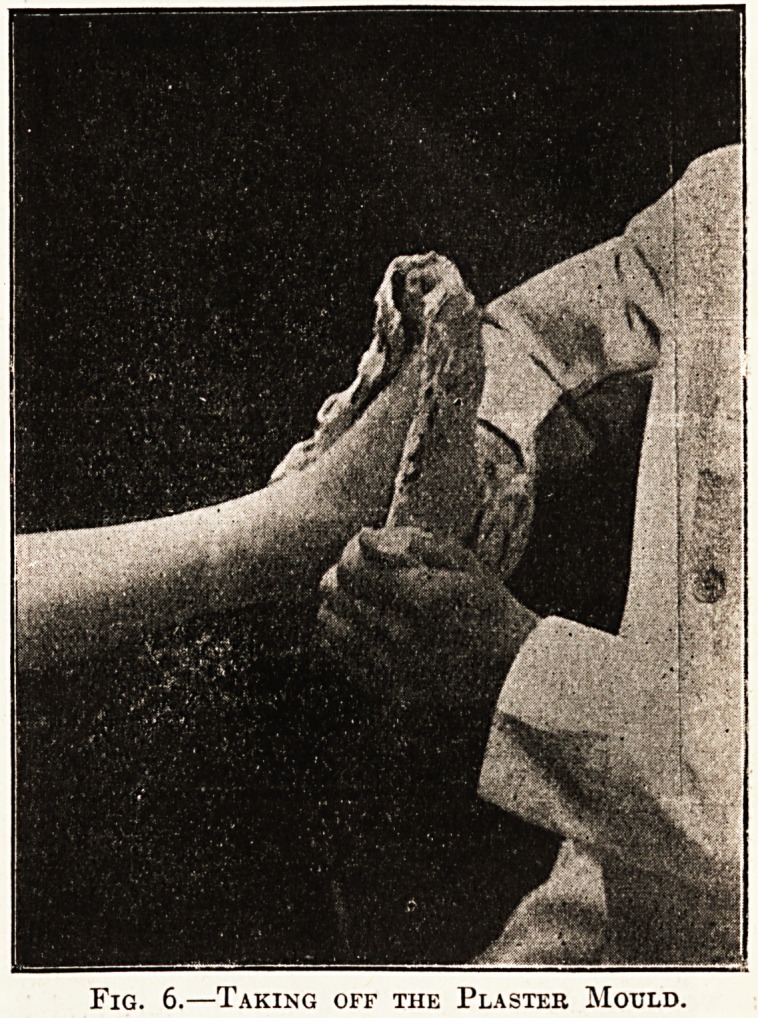# The Orthopædic Department

**Published:** 1914-06-06

**Authors:** 


### THE ORTHOPÆDIC DEPARTMENT.*

### VIII. The Plaster Room (*continued*).

### MODERN METHODS IN CELLULOID WORK.

As the next illustration of the kind of work nowadays carried out in the plaster room of a modern orthopædic department, we may take the technique in the treatment of an uncomplicated case of flat foot. The patient again is a child, since it is only in comparatively young patients that the surgeon meets with a case of uncomplicated deformity of the arch. The deformity is bilateral and symmetrical; were it otherwise, the case would scarcely be uncomplicated, since unilateral flattening of the arch causes a corresponding droop on the side of the deformity, resulting in scoliosis, with the

Fig. 1.—Flat Feet in a Boy of Twelve.

curve opposite to the side on which the flat foot exists.

As a preliminary to treatment, it is necessary to have a record of the degree and extent of the flattening of the arches. Actual measurements are sometimes taken; but it is troublesome and inconvenient to arrange them on a uniform scale. It is, therefore, best to take records of a type that will be more or less uniform, and will at the same time admit of comparison with other records and serve as a basis for future comparison with records taken later on to show the progress of the case. Graphic impressions and photographs constitute by far the best records available. We have already stated in a former article that the orthopædic department should be in direct communication and co-partnership with other departments. One of these departments is the photographic department. Some Continental departments have carried this co-partnership so far that all patients, as a matter of routine, are sent to the photographic department for a graphic record of their deformities; in this way a very valuable series of photographs has been collected, which are highly useful to the workers in the department.

#### Gaits on the Bioscope.

Lately, moreover, the cinematograph has also been extensively used at the Berlin clinic; it gives a very faithful record of the various gaits met with in patients that frequent the orthopædic department, and excellent accounts of the Berlin methods

Fig. 2a. Normal foot impression showing well-formed arch.

Fig. 2b. Impression of flat foot.

in the articles are contributed by Fraenkel to the German *Zeitschrift f. Orthopädie*.

Here we may simply state that the patient is sent to the photographic department, with a request that the feet should be photographed so as to show the flattening of the arch. Obviously, more than one position and various exposures are required to obtain this record; we cannot give a full series of any one such record here, but the annexed photograph shows one of the positions. It will be noted that the child stands with both feet planted firmly on the chair, and the toes slightly turned out; the flattening of both arches is well shown, and the relatively long, "lanky" foot is equally plainly shown. Other positions should show form of the arch when patient stands on his toes, on his heels, and on the outer side of the foot, and front and back photographs should be taken. A complete photo-

* Previous articles appeared on November 1, 15, 29, December 13, 1913, January 24, and May 2 and 23, 1914.

graphic record, although not always necessary, is often of very great use to the surgeon, and serves as a useful basis for comparison.

The next step is to take what is called a perchloride impression of the sole of the foot. To do this the patient's foot is lightly brushed over with

Fig. 3.—An Attempt to Restore the Arch.

a weak solution of tannic acid in water, and he is directed to place his foot squarely upon a sheet of paper which has been brushed over with a fairly strong solution of perchloride of iron. A number of such iron-coated papers must be kept in stock for use when required. Some surgeons use a felt block impregnated with the tannic acid solution, on which the patient places his foot before putting it on the iron-coated paper; when many impressions are to be taken, this method is cleanly and saves time, but for occasional impressions the other method is quite as good.

### The Records Explained.

If the little operation is carefully performed, a permanent impression of the sole of the foot is obtained, which serves as a record, and may be filed with the photographs and clinical notes of the case. The illustrations on page 262 show two of these records. No. 2a shows the normal arch of a child's foot; it will be noticed that the inner edge of the sole hardly touches the paper. No. 2b shows flattening of the arch, the inner edge of the sole being pressed upon the paper and leaving a blurred impression; the extent of such blurring shows the degree of flattening present.

Having now obtained these two records, the question of treatment has to be considered. Exercises are of comparatively little value in the case of the boy whose feet are shown in the first photograph; it is necessary to make a permanent inset for both feet in his case. An ordinary Whitman's brace is hardly suitable; a proper inset must be accurately modelled to suit the foot and to support the arch. To obtain such an inset it is necessary to take a cast of the child's foot, from which the inset may afterwards be modelled in celluloid or brass.

### Methods of the Operator.

Each foot is treated separately, and the method is practically identical for both. The surgeon lightly powders the foot with chalk or boracic acid, lays a small strip of aluminium over the dorsum of the foot, and then rapidly bandages the foot in plaster, covering both ankle and toes, as is shown in the photograph. Before the plaster bandage is applied, however, he lays over the aluminium strip a narrow fret or Gigli saw, which serves to cut open the plaster mould afterwards. While the plaster is still wet the operator takes the foot in both hands, and by manipulation tries to correct the deformity as much as possible; in other words, he tries to restore the arch temporarily, and to let the plaster set round this corrected instep. When the plaster is finally set the saw is rapidly moved up and down, cutting from below upwards, and the plaster mould of the foot is taken off, care being taken not to disturb the correction. A similar mould is taken from the other foot. The two moulds are now allowed to dry completely, are wrapped round with

Fig. 4.—Wasting of Muscles on Inside of Foot.

bandages so as to close any small holes which may be in them, and liquid plaster is poured into them. When this has set, generally after a couple of hours, the outside mould is removed, and a complete cast of the child's feet obtained. This cast is placed upside down on the working bench, fixed in a cast support, and on its instep the inset, or brace, is modelled.

The basis of such an inset is a thin strip of cork, such as a thin cork sole, which is accurately adjusted to the inequalities of the instep and retained in position with tapes, which are tightly wound round it. A solution of celluloid is now made by placing handfuls of celluloid flakes in a wide-mouthed jar containing acetone. The flakes are stirred round with a glass rod until a uniform paste is obtained. With an ordinary painter's brush this paste is now smeared on the cork inset, several layers being applied, until the required thickness, varying from a quarter to an eighth of an inch, is obtained. A few minutes must be allowed for each layer to dry before the other is put on; the solution rapidly evaporates, leaving the celluloid deposited smoothly on the cork. When the whole has hardened sufficiently the inset is trimmed smooth round the edges while still on the cast. Finally, the tapes of the dorsum of the cast are cut through and the inset removed. It now has to be finished, which is done by further trimming down the edges, so that it can easily be slipped into an ordinary boot, and by pasting, with celluloid paste, a thin lining of felt or leather to the upper part. The inset is now completed, and may be tried. Generally it will be found that some further trimming is desirable to make it comfortable for the wearer, but a properly made celluloid inset is exceedingly comfortable, and is far superior to the ready-made Whitman's braces sold by the instrument-makers. It is also necessary to strengthen the arch of the inset by a little cork buttress, which is pasted on with celluloid solution. Such insets last, with ordinary wear, from

Fig. 5.—Correcting the Position in Plaster. (Notice the Gigli saw, A.)

six months to a year, and are made at a cost of from one shilling to one-and-six per pair. With a little practice and experience the surgeon can easily make them himself, or as easily train an assistant or nurse to manufacture them. At Professor Spitzy's private clinic, where such insets have been used for many years, their manufacture is entirely in the hands of the attendants, the Professor himself only taking the moulds and correcting the position of the feet.

The celluloid solution can be used for a variety of other purposes, and serves admirably for the

Fig. 6.—Taking off the Plaster Mould.

manufacture of permanent splints for arm and lower leg cases, and also for jackets and neck casings. For these a basis of cloth or canvas is required, on which the celluloid solution is brushed.

## Figures and Tables

**Fig. 1. f1:**
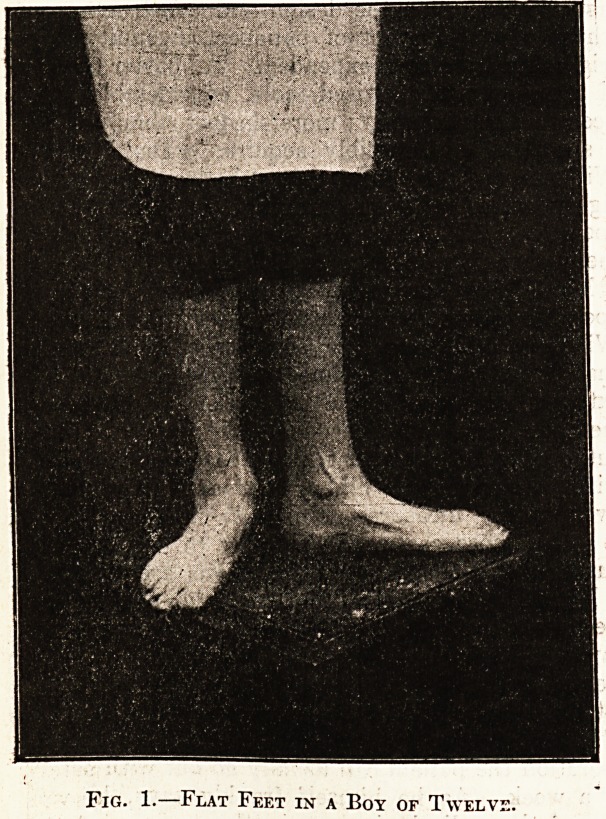


**Fig. 2a. Fig. 2b. f2:**
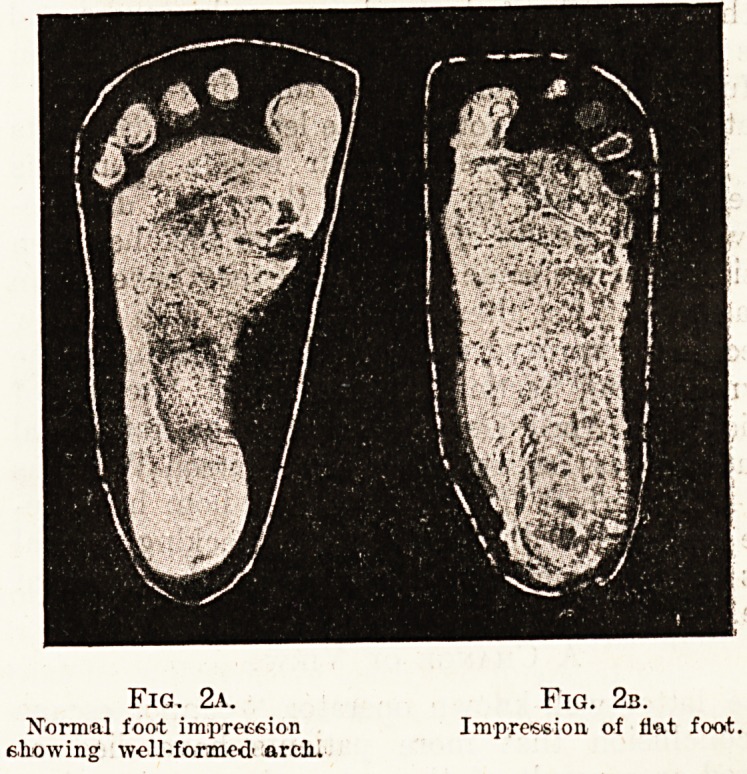


**Fig. 3. f3:**
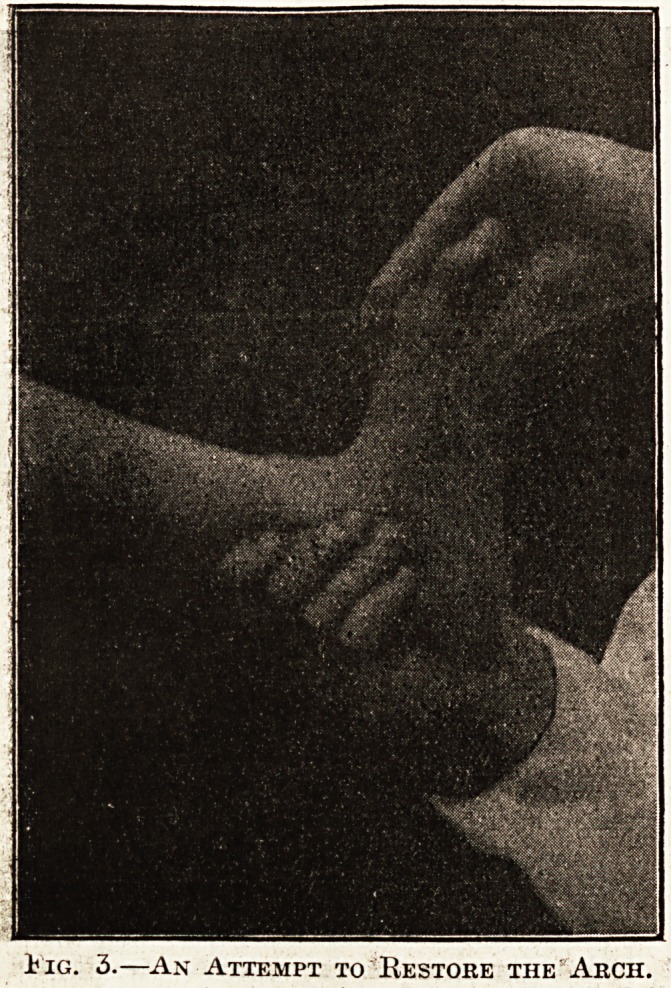


**Fig. 4. f4:**
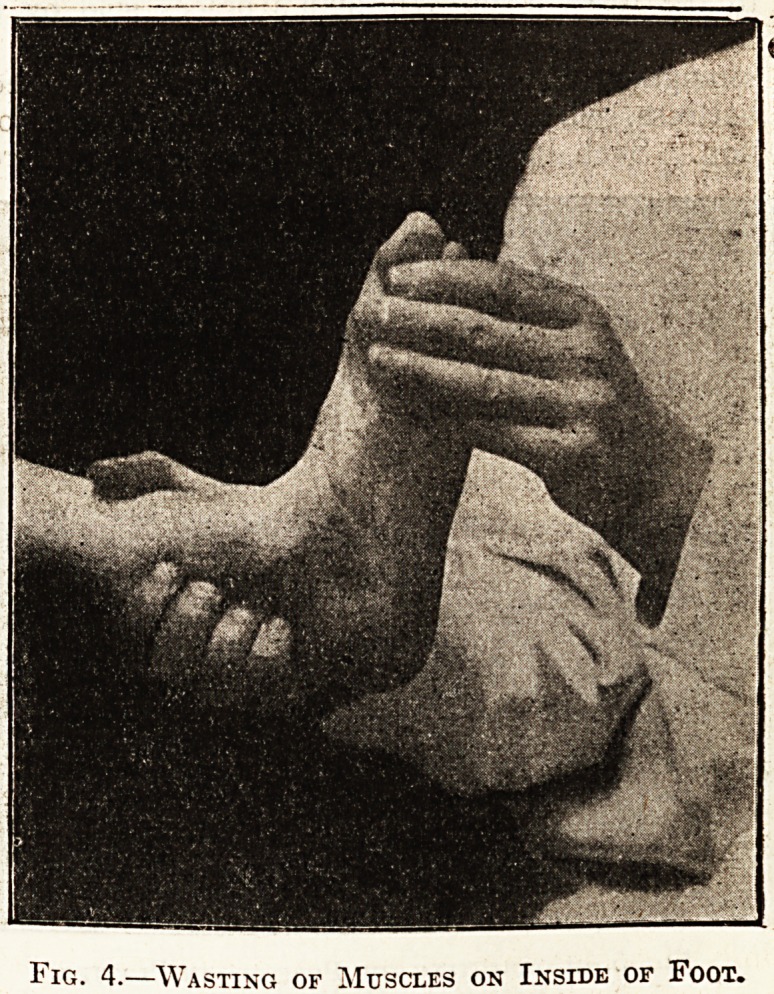


**Fig. 5. f5:**
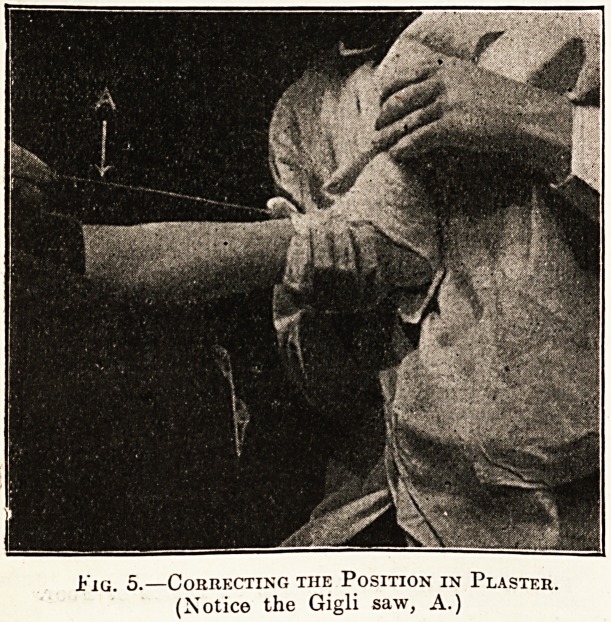


**Fig. 6. f6:**